# Understanding and combating age-related muscle weakness: MYOAGE challenge

**DOI:** 10.1007/s10522-013-9438-3

**Published:** 2013-06-23

**Authors:** Gillian Butler-Browne, Jamie McPhee, Vincent Mouly, Anton Ottavi

**Affiliations:** 1 Université Pierre et Marie Curie, Paris 6, UM76, Institut de Myologie, 47, bd de l’Hôpital, Paris, 75013 France; 2 Inserm, U974, 47, bd de l’Hôpital, Paris, 75013 France; 3 CNRS, UMR7215, Bâtiment Babinski, GH Pitié Salpêtrière, 47, bd de l’Hôpital, Paris, 75013 France; 4 Institute for Biomedical Research into Human Movement and Health, Manchester Metropolitan University, John Dalton Building, Oxford Road, Manchester, M1 5GD UK; 5 Inserm Trasfert, Hôpital du Vinatier, Bat. 452b, 95 Bd Pinel, Bron, 69500 France

Ageing is a hot topic due to the changing demography of developed countries that have seen life expectancy reach an all-time high. The percentage of the population made up of people aged over 65 years increased in all European Union (EU) member countries between 1985 and 2011 and is projected to further increase in all EU countries over the next 20 years. The continuing improvement in mortality rates is an achievement to be celebrated, but we must not overlook the problem that most people aged over 65 years report long-standing illness or disability that reduces their quality of life and restricts their ability to be economically or socially active. Among the many comorbidities of ageing, musculoskeletal dysfunctions are the most common. Thus, a major challenge to researchers, government and, in fact, society at large, is to find ways to preserve health and vitality into older age. In the elderly, muscles become atrophic (loss in muscle mass) and weaker (loss in muscle force), more susceptible to damage and regenerate and recover more slowly than was the case in their youth. Understanding and combating age-related muscle weakness requires a precise definition of the elderly population in terms of mobility and repair capacity, based upon assessment and identification of both physiological and molecular indicators and integration of this data to replace the fragmented and dispersed knowledge that we have of age related muscle weakness. Ageing is a general process of the organism, in which a decline in the proliferation of the progenitors, a lower income of nutrients through vasculature as well as a decreased efficiency of the cellular machinery to metabolize these nutrients, and a less effective dialogue with the other systems participating to the muscle function, result finally in muscle weakness and frailty. Muscle ageing is thus necessarily a multi-component process which will involve as targets the muscle cells, the inflammatory process that increases with aging, as well as weaker tendons and less effective control by the nerves. Combating muscle weakness requires an integrated multi-disciplinary approach that gathers expertise from gerontologists, epidemiologists, cellular and molecular biologists and physiologists. This special issue of *Biogerontology* features original articles and review papers from the MYOAGE study centred on the topic of skeletal muscle ageing. We explore the causes and consequence of reduced muscle mass leading to sarcopenia, muscle weakness and mobility limitations, in order to decipher some potential targets on which to act to allow a “better ageing” for the elderly population.

The MYOAGE study was an EU-FP7-funded large-scale project involving 19 leading academic or industry-based research teams from 10 EU countries. The project encompassed an integrative approach of epidemiological, physiological, cell and molecular and genetic investigations of patients suffering chronic disease involving muscle weakness, compared to healthy humans and master athletes with developed muscle strength, as well as rodent and cell culture models. The overall aim was to investigate the contribution of age-related changes to muscle mass, contractile characteristics and neural control in relation to mobility limitations in healthy young and older adults using standardised assessments. This multi-disciplinary pan-European approach has allowed us to define healthy and impaired muscle ageing and to identify new biomarkers and risk factors of muscle weakness in older age. The project was organised in three main aims: *Collect, collate and combat*. These aims were achieved through eight work packages (WPs) with a scientific focus, 1 WP specifically addressing ethical issues and 2 WPs dealing with overall study management and training of emerging scientists. In this special issue of *Biogerontology* we report the detailed methodology and phenotype data collected from 504 young and older healthy men and women. Biological material (muscle biopsies, muscle cell cultures, blood and serum samples) were collected from these well characterised young and old sedentary and active subjects and was used by all research groups to try to correlate the age related physiological modifications to cellular and molecular mechanisms. The different chapters in this issue highlight some of the findings of this study.

One striking theme to emerge from the integration of results from this project was that findings from animal models are not necessarily an indication of human ageing. Detailed examination of human tissue offered several “surprise” findings and emphasized the role of an “ageing environment” on muscle function and regenerative capacity. However, there was considerable “heterogeneity” between samples that often made it difficult to reach final conclusions about the respective role of the molecular processes in healthy or diseased ageing. This combined effort should be followed up in larger human trials.

The MYOAGE ethos was to promote multi-disciplinary, collaborative work between established scientists and industry as well as the development and mentorship of emerging young scientists. The results of this work provide a detailed insight into “normal, healthy” ageing as well as disease and link whole-body function and systems-biology to the structure and function of the neuromuscular systems and the molecular characteristics of skeletal muscle. The work will be used to define guidelines for public health promotion in older age. All data have been retained in a database. A biobank including blood and muscle tissue samples is being maintained and we welcome opportunities to collaborate and further develop our understanding of the impact of ageing on neuromuscular function and health. Finally, several workshops were organized for young students and scientists, thus paving the way to a body of confirmed scientists within the field of muscle ageing.

In conclusion, the concerted research projects conducted within MYOAGE, and which associated clinicians, cell and molecular biologists and physiologists, most of the members having multiple expertise, confirmed some data described on animal models, but also infirmed others, thus designing a new landscape representing human muscle ageing. This standardized description of physiological, cellular and molecular mechanisms involved will allow us to consider ageing of each organ as integrated into the ageing organism in order to identify relevant targets to provide to the EU population conditions for “healthy ageing”.

